# Improvement of Scratch and Wear Resistance of Polymers by Fillers Including Nanofillers

**DOI:** 10.3390/nano7030066

**Published:** 2017-03-16

**Authors:** Witold Brostow, Haley E. Hagg Lobland, Nathalie Hnatchuk, Jose M. Perez

**Affiliations:** 1 Department of Materials Science and Engineering, University of North Texas, 3940 North Elm Street, Denton, TX 76207, USA; haleylobland@gmail.com (H.E.H.L.); hnatnm@gmail.com (N.H.); jose.perez@unt.edu (J.M.P.); 2 Department of Physics, University of North Texas, 3940 North Elm Street, Denton, TX 76207, USA

**Keywords:** scratching of polymers, polymer wear, polymer + nanofiller composites, polymer tribology

## Abstract

Polymers have lower resistance to scratching and wear than metals. Liquid lubricants work well for metals but not for polymers nor for polymer-based composites (PBCs). We review approaches for improvement of tribological properties of polymers based on inclusion of fillers. The fillers can be metallic or ceramic—with obvious consequences for electrical resistivity of the composites. Distinctions between effectiveness of micro- versus nano-particles are analyzed. For example, aluminum nanoparticles as filler are more effective for property improvement than microparticles at the same overall volumetric concentration. Prevention of local agglomeration of filler particles is discussed along with a technique to verify the prevention.

## 1. Scope

There is an ongoing process of replacement of metal parts by polymer-based composites (PBCs) in automotive, aviation, and aerospace industries (this list of industries is not claimed to be complete). The advantages are well known: less and cheaper maintenance; less noise (examples: Boeing Dreamliner, Aurora D8); and lower overall weight, which in the case of automobiles and planes lowers fuel consumption. Growth of the electronics industry also seems to be accompanied by increased use of polymers or of polymer-based composites. The replacement process is slowed down by:

- Need for PBCs with higher service temperatures. Metal service temperatures are much higher than those of PBCs. This situation behooves us to develop PBCs with higher and, if possible, predictable glass transition regions [1].
- Need for PBCs with better mechanical properties. Strength, toughness, etc. of metals typically exceed those of PBCs.
- Need for higher scratch resistance in PBCs. Polymers are typically scratched more easily than metals.
- Need for PBCs with higher wear resistance. Wear occurs as the unwanted loss of material from solid surfaces due to mechanical interactions. Though liquid lubricants can be used to increase wear resistance of metals, that approach is not widely useful for PBCs because of polymer swelling.

A comprehensive review of all these issues could be voluminous. In this article, we must necessarily note some important thermophysical and mechanical properties of PBCs, but the emphasis is on what we know and how we can improve tribological properties, namely scratch resistance, wear, and friction, of PBCs. As pointed out by Ernest Rabinowicz of MIT [2], annual losses to industry resulting from insufficient tribological expertise run into many millions of dollars. In many cases, parts and components that serve their purposes in dynamic contact with other parts require periodic replacement due to wear when they are otherwise mechanically sound. Such replacement is often palliative only, when a longer lasting component would prove more efficient and less costly. Moreover, as discussed by Zambelli and Vincent [3], the energy needed to maintain movement of the worn parts is higher than for “fresh” parts. 

Here, we review methods of improving tribological properties of polymers—current and potential ones. The common denominator for many of these methods is the use of fillers. Fillers may be comprised of micro- or nano-sized particles. Our PBCs are sometimes called ‘hybrids’ since they defy the traditional classification of materials into inorganic and organic; here we describe materials that have organic polymers as matrices and inorganic (metal or ceramic) fillers. 

## 2. Spatial Distribution of the Filler

Some engineers and scientists consider all matrix + filler composites ‘treacherous’. The reason is potential local agglomeration of the filler particles; the properties at such a location are much worse than those of the neat polymer without filler. Thus, before doing anything else, one needs to verify uniformity—or otherwise—of the spatial distribution of particles in the matrix. A technique for such verification has been developed [4]; it involves two different kinds of equipment. First, a focused ion beam (FIB) is used to ‘cut out’ a parallelepiped in a specimen of the composite. In the case described in reference [4], the milling out of the specified volume was performed with Ga^+^ ions at a 30 keV acceleration voltage. It was found afterward that gallium ions deposited on the new surface hampered visualization; however, passing a low beam current of 3 nA over the surface restored the visibility. Another complication of the FIB process was heating caused by the Ga^+^ beam. This problem was eliminated by changing the overlap in the passes done by the beam from 50% to 0%. The FIB use was complemented by scanning electron microscopy (SEM)—a technique well explained by Gedde [5] and also by Michler [6]. SEM was performed before, during, and after ion milling since effects of that process needed to be evaluated, too.

**Figure 1:**
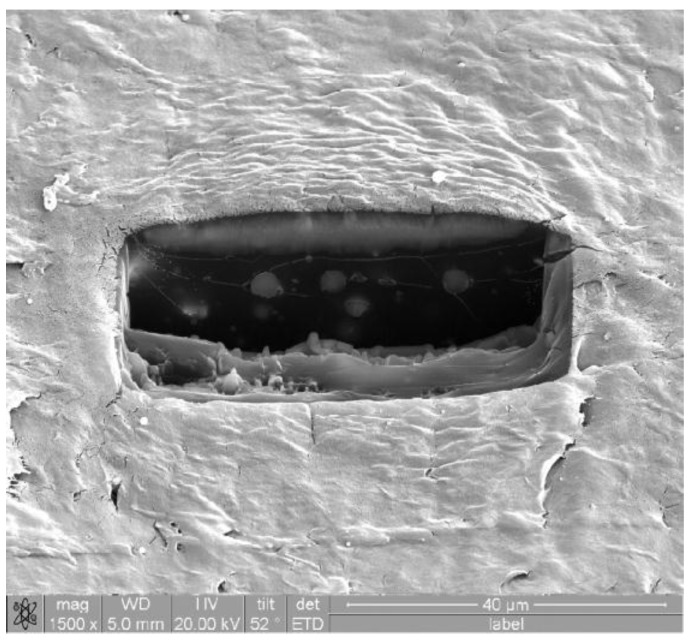
Scanning electron microscopy (SEM) micrograph of a polyethylene (LDPE) + Al composite after milling; the bar in the Figure is 40 µm.

The composites described in [4] consisted of low density polyethylene (LDPE) with aluminum particles—approximately spherical, with the average diameter of 1 µm. One of the scanning electron micrographs obtained is shown in Figure 1. We see no agglomeration of filler particles on the walls of the parallelpiped. Actually, particle size is not an obstacle in verification of agglomeration; smaller particles, alone or in agglomerates, can be visualized since SEM can resolve details such as 10 nm [5,6]. The FIB/SEM technique developed in 2007 has been used since for various composites. 

Needless to say, not all filler particles have a natural tendency for uniform distribution in a polymeric matrix. Olsson and his colleagues studied spatial distribution of cobalt ferrite nanoparticles (Co*_x_*Fe_3−*x*_O_4_, *x* ≈ 1) in an epoxy. First, they developed a method of synthesis of such particles [7,8] with a diameter of ≈ 50 nm. Then they applied three kinds of coatings: 3-glycidoxypropyl- (GPTMS), aminopropyl-(APTMS), or methyl-silsesquioxane (MTMS). Three types of behavior were observed [9]. MTMS-coated ferrite particles with a coating thickness of 3 nm agglomerated in the composite without sedimentation. APTMS-coated particles agglomerated and underwent sedimentation. 50 nm thick GPTMS coatings provided the desired effect: no agglomeration or sedimentation. 

## 3. Metal Fillers

Let us take a closer look at several different polymer + metal composites. Microparticles of Al, Ag, and Ni have been used in turn as fillers in LDPE and also in Hytrel^®^ (DuPont, Wilmington, DE, USA), which is a polyester used as a thermoplastic elastomer [10]. Dynamic and static friction of the composites were determined with stainless steel and Teflon^®^ (Chemours, Wilmington, DE, USA) (tetrafluoroethylene) as sliding counterpart surfaces; tensile properties were determined as well [10]. Similarly, nanocomposites of LDPE with either Al or Ag nanoparticles were created, and wear was determined for those with a pin-on-disk tribometer, with carbon nitride pin balls [11]. Wear and dynamic friction determination using a pin-on-disk tribometer is discussed elsewhere by some of us [12]. 

**Figure 2:**
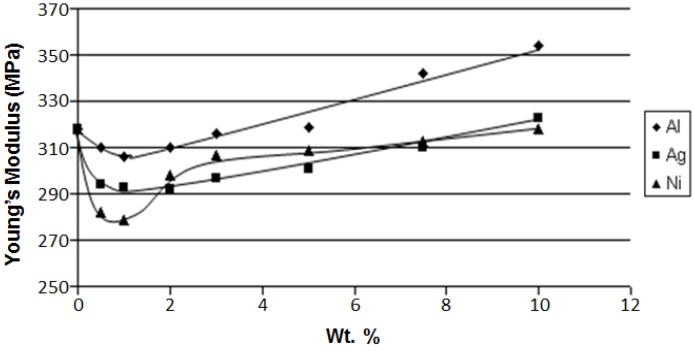
Tensile modulus of Hytrel as a function of the metal micropowder concentration. Reprinted from [10] with permission. Copyright Society of Plastics Engineers, 2008.

We consider first tensile and crack propagation results reported in [10]. Addition of Al, Ag, or Ni microparticles to both LDPE and Hytrel up to 10 wt % resulted in diagrams that exhibit a minima of modulus E as a function of the filler concentration. It is observed that at various filler concentrations, the modulus may be higher or lower than that of the unfilled neat polymer. For LDPE, at 10% filler, the tensile modulus for all three filler types is lower than the tensile modulus for neat LDPE. By contrast, for Hytrel, the E values at 10% concentration are comparable to neat Hytrel for silver and nickel but much higher for aluminum; see Figure 2. 

Apparently, at low concentrations, each filler lowers the matrix cohesion for both LDPE and Hytrel. This effect is stronger than the positive effect of inclusion of the reinforcement. At higher concentrations, the situation becomes reversed, that is the reinforcing effect of the filler now dominates the modulus. Hence the minima in the curves seen in Figure 2. Backscattered electron SEM images for LDPE + 10% Ni show an interesting phenomenon: crack arrest. The images reveal that crack propagation is clearly halted as cracks approach the Ni particles. Thus, one expects that a higher energy will be needed for fracture of the composite than for neat LDPE. There is again a contrast between polyethylene and the elastomer Hytrel; crack arrest by the filler is not seen in the latter. 

**Figure 3:**
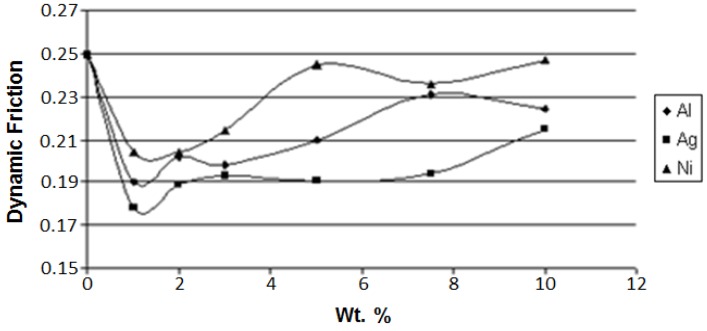
Dynamic friction of the polyethylene (PE)-based composites with metal microfillers against steel as the sliding surface. Reprinted from [10] with permission. Copyright Society of Plastics Engineers, 2008.

Continuing with our analysis of the effects of metal fillers in polymer-based composites, we consider now dynamic friction of these composites and provide a diagram for LDPE-based composites in Figure 3. 

We see in Figure 3 for all three metal fillers a reduction of friction to a minimum followed by an increase at higher filler concentrations. The minima all lie at approximately 1 wt %, hence the costs of adding a metal filler to achieve lower friction are not excessive. Such minima are also seen on the diagrams of dynamic friction of two out of three kinds of LDPE-based composites against Teflon, namely for aluminum and silver. For nickel, there is an initial friction lowering, while above 3 wt % of Ni the friction simply does not change with increasing nickel concentration. 

We now need to explain such friction minima. As seen in Figure 1 and also in SEM micrographs in [10,11], we are dealing with ductile polymers and composites. Protrusions on their surfaces contribute to friction. The micrographs of wear tracks show more protrusions in pure LDPE than in the composites. Thus, addition of metals initially lowers the number of protrusions per unit area such as 1 cm^2^. Thus, one gets a lower effective contact area—as contrasted with the large nominal area. However, when the number of filler particles increases, the real contact area increases also. 

**Figure 4:**
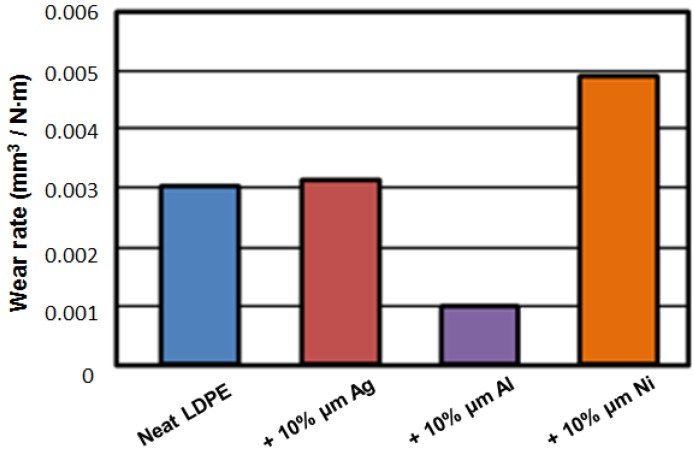
Wear rates for neat LDPE and LDPE-based microcomposites against carbon nitride pin balls. Labels indicate the percentage of metal micropowder in LDPE.

There is an interesting question: given the same volumetric fraction of the fillers, are microparticles or nanoparticles more effective in property improvement? Results reported in [11] will be used for that discussion. Here, LDPE was the matrix; microsize particles were of Al, Ag, and Ni; nanoparticles were of Al and Ag. Wear rates for microsize fillers in LDPE determined against carbon nitride pin balls are shown in Figure 4. Carbon nitride pins were used because of the high hardness of carbon nitride.

The results in Figure 4 show fundamental differences between effects of different metal particles. Silver addition results in a slight, but negligible, increase of the wear rate. Aluminum addition results in a significant decrease of the wear rate. Nickel addition causes a large increase of the wear rate.

Myshkin and his colleagues in Homel [13] considered the factors affecting wear. They concluded that the main factors are: interfacial bonds and the resulting adhesion (including the type and strength of the bonds); real contact area (related among other things to debris formation); and shear and other deformation phenomena at and around the contact points. The main mechanism of wear in neat LDPE is deformation; in composites with Ag it is adhesion, but the resulting wear rates are close to each other. For Ni-containing microcomposites, debris formation is the main wear mechanism, with the debris particles in the form of rolls. For Al composites, the main wear mechanism is believed to be the three-body abrasion, which apparently ‘levels’ the protrusions on the surface of the polymer and so decreases the real contact area.

**Figure 5:**
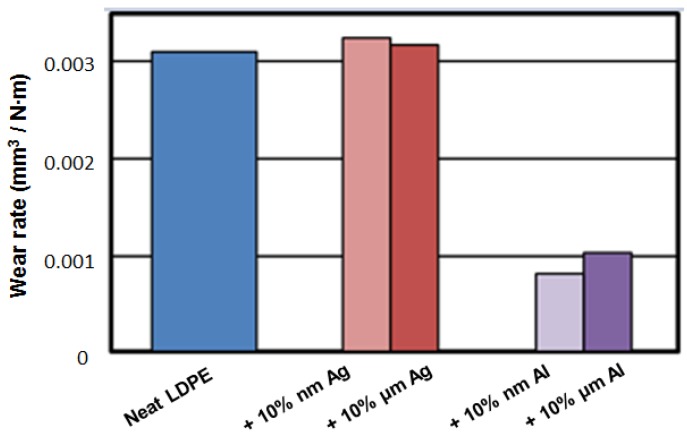
Wear rates for LDPE and its composites—containing nano- or micro- particles—against carbon nitride pin balls under the load of 7.0 N.

We now proceed to the comparison of effects of micro- and nano-particles of metals [11]; see Figure 5. We see in Figure 5 that the addition of silver causes insignificant increase of the wear rates, both for nano- and micro-particles, with slightly higher values for the former. With the addition of aluminum, we see a dramatic lowering of the wear rates as compared with neat PE. The lowering effect is somewhat stronger for nanoparticles. We recall that the dominant effect of wear in aluminum-containing composites is three-body abrasion resulting in leveling of the protrusions. The volumetric concentration of the Al particles is the same for both sizes of the particles. Apparently, a larger number of smaller particles produces a stronger leveling effect.

Inspecting Figure 2, Figure 3, Figure 4 and Figure 5 we also see connections between various properties. At 10 wt % of the filler, aluminum has the highest modulus (Figure 2). In Figure 3, we see that the aluminum filler provides the lowest dynamic friction. In Figure 4 and Figure 5, we see that aluminum also provides the lowest wear rates by far, this for both micro- and nano-size fillers. Thus, we have here a connection between friction and wear rate. Svahn and coworkers in Uppsala have studied that connection for tungsten- or chromium-containing amorphous carbon coatings on stainless steel machine elements [14]. Steel plates were grinded to a different extent—leading to different surface roughness values—and then the coatings applied. Dynamic friction was determined with a pin-on-disk tribometer with steel pins. As expected, higher roughness resulted in higher dynamic friction values. However, the wear rate for the tungsten containing coating was independent of the roughness. In contrast, for the chromium containing coatings, the roughest coatings wear up to six times more than the smoothest ones. An at-least partial explanation is based on the presence of fatigue pits in wear scars of the chromium-containing coatings deposited on rough substrates. No such pits appeared on the tungsten-containing coatings. 

Given the consequences of inclusion of nickel nanoparticles seen in Figure 4, in a separate study such particles were added to a thermoplastic elastomer (TPE) consisting of ethylene-propylene-diene rubber and modified with metallic diacrylate co-agent [15]. The combination of FIB and SEM discussed above has shown that melt mixing at 100 °C has resulted in a fairly uniform particle distribution. In contrast, such mixing at 160 °C, and necessarily at lower viscosity, facilitated nickel particle agglomerations. Brittleness B as defined in 2006 in [16] was also measured. While addition of Ni increases brittleness B, and so does vulcanization, a combination of both these treatments lowers B. Apparently, some Ni particles go into existing free volume spaces in vulcanized materials—thus enhancing mechanical properties, including the dynamic storage modulus E’. At the same time, other filler particles are able to create new free volume pockets—increasing the elongation at break ε_b_. Thus, Ni particles at both kinds of locations provide lower values of B. We recall that B is inversely proportional to ε_b_ and also to viscoelastic recovery in scratch-resistance testing [16]. That recovery is manifested in the bottom of the groove going up within no more than two minutes. 

While gold nanoparticles are not a typical filler kind, dynamic friction and wear were studied for polystyrene (PS) plus such particles [17]. First, gold particles with a diameter of ≈ 15 nm were prepared by laser ablation in a liquid environment, mixed with the polymer under a solvent, then the solvent was evaporated and specimens were prepared by compression molding. Also, here the FIB/SEM check on filler dispersion uniformity was performed—with positive results. 2000 revolutions were made on the pin-and-disk machine with silicon nitride as the pin material. Three types of response were discerned from an analysis of the wear tracks: brittle response of PS resulting in formation of debris shaped approximately as platelets; abrasion of metal particles by the pin, resulting in formation of irregularly shaped debris particles; and protection of the surface against abrasion by the filler particles. The magnitude of the last effect increases along with increasing volumetric concentration of Au particles. Above 0.1 wt % of gold particles, both dynamic friction and wear loss volume went down. Since PS is an unusually brittle material [16], introduction of gold nanoparticles into other polymers should also lower dynamic friction and wear. 

One more application of gold nanoparticles in a polymer matrix will be mentioned. A combined group at Windsor/Ontario and São Carlos has embedded such particles in the biopolymer chitosan [18]. Self-supporting thin films were obtained. Such films might have a number of applications, including biosensors. To demonstrate this particular application, chitosan plus gold nanoparticles composite was used in trace analysis via surface enhanced Raman scattering. 

To conclude this section, let us mention polymer + metal nanocomposites that provide antibacterial activity [19]. The polymer used was chondroitin sulfate which is a sulfated glycosaminoglycan consisting of a chain of alternating sugars. Silver nanoparticles were obtained by reduction of AgNO_3_ in aqueous solution of the sulfate. Reduction was performed by either thermal treatments at 80 and 90 °C or else by UV irradiation. Then the samples were placed in insulin syringes, cooled to the liquid nitrogen temperature, and subsequently freeze-dried to produce 3-dimensional scaffolds. The antimicrobial activity of the scaffolds was tested against *Escherichia coli*. The minimum inhibitory concentration (MIC) for the scaffolds turned out to be ≈ 6 ppm.

## 4. Ceramic Fillers

While the results already noted above suggest the usefulness of using filler particles to improve tribological properties, there is still much attention devoted to the use of such fillers to provide mechanical reinforcement. Thus, Adhikari and his colleagues introduced organically modified alumina nanoparticles into polystyrene-polybutadiene-based triblock copolymer (SBS) [20]. The addition of 5 wt % boehmite enhanced the tensile yield stress without lowering the strain at break. Boehmite is an aluminum oxide hydroxide AlO(OH). Addition of higher boehmite concentrations, however, resulted in filler agglomeration—discussed by us in Section 2—with the aggregate sizes exceeding 100 nm. 

In related work, boehmite was treated with sulfonic acid-based surfactants, again used as a filler for SBS, and several mechanical properties were determined [21]. With both nanoindentation hardness h_nanoident_ and Vickers hardness h_Vickers_ determined, it turned out that the ratio of these two hardnesses is a constant for all SBS-containing composites. 

**Figure 6:**
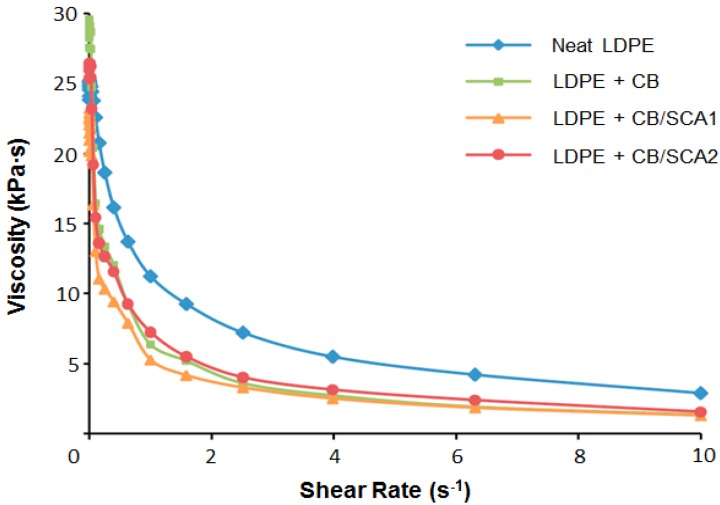
Viscosity as a function of shear rate LDPE and its composites with heated commercial boehmite (CB), without and with silane coupling agents (SCA1, SCA2); from [22]. Reprinted with permission. Copyright Society of Plastics Engineers, 2010.

With processing as a necessary preparation for making samples for testing, one often finds that the filler enhances the viscosity of the molten polymer. However, boehmite added to LDPE results in a viscosity decrease [22]; see Figure 6. While in high temperature polymers (HTPs) treated with a variety of ceramic fillers, we see “everything” [23]: viscosities that are higher, lower, and comparable to the viscosities of the neat polymers. 

**Figure 7:**
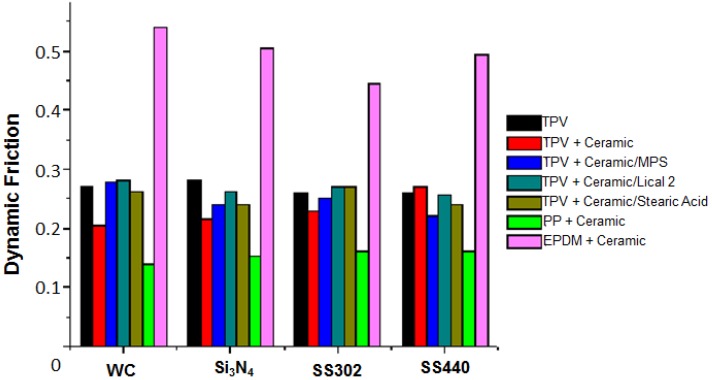
Friction values for thermoplastic vulcanizate, its constituents and composites. Reprinted from [24] with permission. Copyright Society of Chemical Industry, 2012.

Some ceramic fillers are characterized as “thermal shock resistant”, apparently surviving temperatures as high as 1400 °C. Microsize such particles were put into a thermoplastic vulcanizate (TPV), namely blends of polypropylene (PP) and ethylene-propylene-diene (EPDM) rubber at the concentration of 5 wt % [24]. The fillers included corundum (α-Al_2_O_3_), mullite (3Al_2_O_3_^.^2SiO_2_), the eutectic of both (a summary formula ≈ 2Al_2_O_3_SiO_2_), modified β-spodumene (with negative thermal expansivity) and stabilized aluminum titanate. The ceramics were prepared by interaction of the minerals in the air atmosphere at 1550 °C temperature for 5 h. Dynamic friction was measured with pin-on-disk tribometry vs. pins of tungsten carbide, Si_3_N_4_, and two kinds of steel, SS302 and SS440. Three coupling agents were used, two of them silanes (SCA and MPS) and one titanate (Lica 12). The results are presented in Figure 7.

In Figure 7, we see that the lowest value of dynamic friction is for PP + Ceramic while the highest is for EPDM + Ceramic. For all counter-surfaces, friction curves of the TPV and its composites fall between the PP + Ceramic and EPDM + Ceramic samples.

Too high of an electric conductivity constitutes a problem for certain applications. One such situation involves polymer-based components used in the distribution of solar energy power; this is particularly the situation in distributing solar power from Africa to northern Europe. To reduce power losses, direct current voltages exceeding 800 V are preferred. If PE alone is used as a cable insulator, local accumulation of charges may lead to heat-associated insulation breakdown. Pallon and coworkers have succeeded in lowering the electric conductivity of PE two orders of magnitude by the inclusion of MgO nanoparticles [25]. An important problem appeared in this case which also appears in other polymer + ceramic filler applications: the polymer is hydrophobic, the filler hydrophilic. In the case just named, surfaces of the MgO nanoparticles were silanized, resulting in formation of silsesquioxane, an organosilicon compound. We have noted in the beginning of Section 2 the importance of avoiding filler particle agglomeration and achieving uniform spatial distribution. The unmodified particles did agglomerate, as seen in SEMicrographs of fracture surfaces. The definition of an agglomerate used in [25] is: a cross-section that is twice that of the diameter of two solitary filler particles, what for MgO means above 132 nm. By contrast, silanized particles were uniformly distributed in the LDPE matrix—as also seen in SEM. 

There is another problem related to the use of PE with MgO nanoparticles as a coating for high voltage DC cables: in humid environments, these particles undergo a conversion to Mg(OH)_2_. A multistage procedure for dealing with this problem has been developed [26]. One in fact starts with Mg(OH)_2_, performs its thermal decomposition at 400 °C, applies a silicone oxide coating which conceals the nanoparticles, and then applies a heat treatment at 1000 °C. MgO particles created in this way show good dispersion in the LDPE matrix, good adhesion to that matrix, and long term resistance to moisture. As compared to neat LDPE, the electric conductivity is reduced by a factor of 30. 

**Figure 8:**
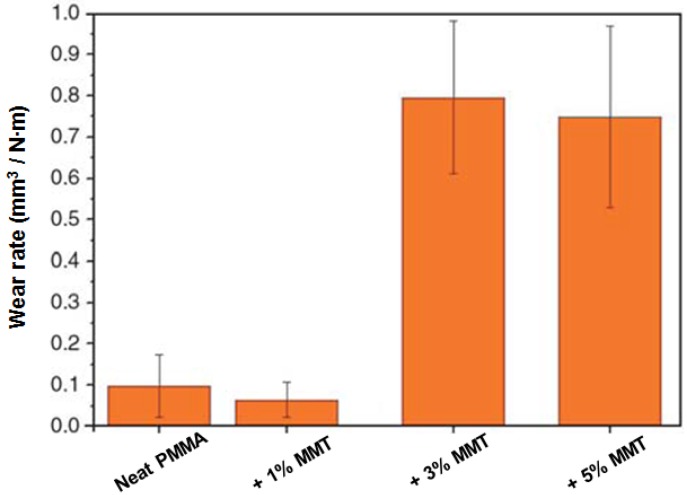
Wear rates for poly(methyl methacrylate) (PMMA) and its composites with MMT. Reprinted from [27] with permission of the copyright holder.

An inexpensive kind of filler sometimes used for improvement of tribological properties of polymers is clay. Wear rates for poly(methyl methacrylate) (PMMA) and its composites with montmorillonite (MMT) Brazilian clay [27] are shown in Figure 8. 

We see in Figure 8 that the wear rate as a function of the clay concentration passes through a minimum at 1.0% clay. Thus, for neat PMMA and PMMA with 1.0% MMT clay, the wear rates are much lower than for the composites with 3.0% and 5.0% of MMT clay. Often the presence of clay decreases the elongation at break ε_b_ of the material. As noted above, ε_b_ is inversely proportional the brittleness [16]. Apparently, the increase of B leads to an increase in wear rate.

Sepiolite, a fibrous clay, was used as a filler in arabinoxylan (RAX) films [28]. RAX is one of hemicelluloses, an abundant kind of heteropolysaccharides. The problem of filler aggregation, noted by us in this article several times, did not appear—possibly because of the hydrogen bonding between sepiolite and arabinoxylan seen in FT-IR spectroscopy. Clear strengthening of the films by sepiolite was seen. RAX alone has the tensile Young’s modulus *E* = 2.3 GPa. Addition of only 5% of sepiolite resulted in the modulus ‘jumping up’ to 4.2 GPa. The addition of plasticizer was also explored. The plasticizer lowered the mechanical properties but it significantly increased ε_b_.

A separate and interesting issue is the filler concentration. As we have seen above, in some cases lower filler concentrations provide better properties than higher ones. Volker Abetz and coworkers [29] created composites of poly (vinyl butural) (PVB) with micron size alumina particles, reaching up to 77 vol. % of the latter. In such composites, filler–filler interactions are important, in addition to the matrix–filler interactions. The filler introduction results in an increase of the storage modulus of PVB by four orders of magnitude. The location of the glass transition region was practically unaffected by the filler and changes of the filler concentrations. 

## 5. Carbon-Containing Composites 

While carbon is classified as an inorganic material, it appears in several forms with a very wide range of properties, hence it seems to deserve a separate section. As discussed by Svahn and coworkers [14], a micron-thick carbon coating can decrease wear by orders of magnitude and maintain a low friction in a non-lubricated contact.

Carbon nanotubes (CNTs) and graphene have been investigated as fillers for enhancing the mechanical, electrical, and tribological properties of polymers. These carbon nanomaterials are lightweight and can be functionalized to improve dispersion and adhesion to the polymer matrix. In particular, functionalized reduced graphene oxide (FRGO) can be easily and inexpensively mass-produced. Graphene itself, a two-dimensional material, applicable in very thin layers, can be used as a self-lubricating solid or else as an additive to lubricating oils [30]. The large surface to volume ratio of these reduced-dimensionality materials means that there is significant interaction between filler and polymer matrix even at low filler concentration, allowing a wide range of mechanical, electrical, and tribological properties to be tailored by varying filler concentration. Polymer composites containing CNTs [31] and graphene [32] fillers have outstanding mechanical and electrical properties. The inclusion of metal nanoparticles in CNTs and graphene results in composites with outstanding electromagnetic absorption and shielding characteristics [33,34].

The volumetric wear and brittleness of hybrids of the thermoplastic elastomer poly(butyl terephthalate)/oxytetramethylene with single wall CNTs (SWCNTs) and multi wall CNTS (MWCTS) show minima as a function of filler concentration [35] while the Young modulus and strain at break show the maxima. The plasticity of the hybrids increases as evidenced by an increase in elongation at break. The plasticization is more significant for hybrids containing SWCNTs than MWCNTs due to SWCNTs having fewer contact points with the matrix per unit surface area and greater flexibility.

**Figure 9:**
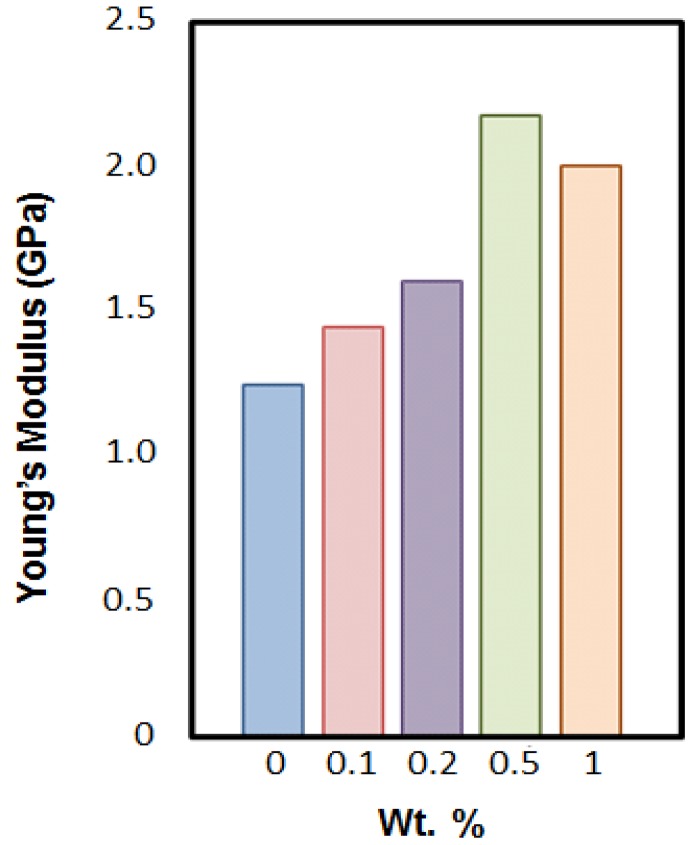
Young’s modulus vs. functionalized reduced graphene oxide (FRGO) concentration for epoxy + FRGO hybrids.

The mechanical and tribological properties of hybrids of diglycidylether of bisphenol-A epoxy with octadecylamine-functionalized reduced graphene oxide (FRGO) show a maximum in the Young’s modulus at 0.5 wt % of FRGO [36], as shown in Figure 9. Also, the tensile strength and strain at break show a maximum and minimum, respectively, at 0.5 wt % FRGO. The glass transition temperature T_g_ which represents the glass transition region [1] has a maximum at 0.5 wt % FRGO as well. For all these properties, the behavior above 0.5% FRGO might be due to filler agglomeration (almost a leitmotiv of this review…). 

**Figure 10:**
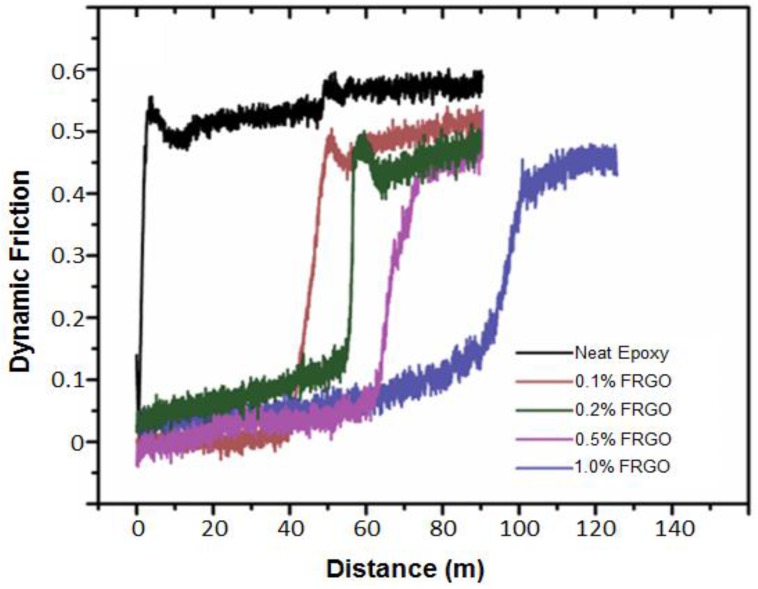
Dynamic friction vs. sliding distance for epoxy and its hybrids with FRGO.

In the same system, the friction as a function of sliding distance, as measured using a pin-on-disc tribometer, displays a regime in which the friction is on the order of five times less than that of the neat epoxy, as shown in Figure 10. This is attributed to a thin transfer film of the hybrid onto the tribometer pin. After a sliding distance of about 50–100 m, the friction returns to a value comparable to that of the neat epoxy, due to removal of the transfer film by residue in the wear track. While the dramatic difference is not seen anymore, all friction values for the hybrids are still lower than for the neat epoxy. 

## 6. Concluding Remarks

As noted in Section 1 citing Rabinowicz [2], increasing tribological expertise is related not only to scientists and engineers trying to excel in the development of new materials, but also to industry economics. Tribology of metals is ‘older’, well advanced, and in many cases liquid lubricants are doing at least a satisfactory job. However, we need to remember the warning by Blau [37]: “…the fundamental origins of sliding resistance are not as clear. In fact, some of the greatest scientists and philosophers have contemplated friction without managing to produce a universal, predictive theory. This striking lack of success is due to the many potential factors that can influence friction in a wide spectrum of physical situations”.

With softer and mechanically weaker polymers, and with the liquid lubricants not well applicable, filler particles provide interesting and useful options. In our review, we tried to provide a balanced view of this area, stressing also difficulties involved in preventing filler particle agglomeration. Again, on the positive side of the ledger, we need to note that filler concentrations providing visible improvement of tribological properties are often low. Figure 8 showing the minimum of wear at 1.0 wt % of inexpensive nanoclay seems pertinent from this point of view.
